# Fast Track Intervention Effects and Mechanisms of Action Through Established Adulthood

**DOI:** 10.1007/s11121-024-01736-0

**Published:** 2024-10-11

**Authors:** George McCabe, Jennifer W. Godwin, W. Andrew Rothenberg, Natalie Goulter, Jennifer E. Lansford, Conduct Problems Prevention Research Group, Karen L. Bierman, John D. Coie, D. Max Crowley, Kenneth A. Dodge, Mark T. Greenberg, John E. Lochman, Robert J. McMahon, Ellen E. Pinderhughes

**Affiliations:** 1 Duke University, Center for Child and Family Policy, Durham, USA; 2 Flinders University, College of Education, Psychology, & Social Work, Flinders Institute for Mental Health & Wellbeing, Adelaide, Australia

**Keywords:** Childhood, Adulthood, Prevention, Intervention, Mechanisms

## Abstract

Early preventive interventions can improve outcomes in childhood, but the most effective interventions can continue to deliver benefits through the life course. The Fast Track intervention, a randomized controlled trial for children at risk of conduct problems, has lowered psychopathology, substance use problems, and criminality and elevated happiness at age 25. However, research has not studied whether the intervention’s effects continue further into established adulthood. In addition, little is known about the mechanisms through which the intervention may affect adult outcomes. We attempted to answer both questions by simultaneously estimating the intervention’s direct effect on adult outcomes at age 31 and the intervention’s indirect effects on those outcomes via interpersonal, intrapersonal, and academic competencies gained through the intervention. Participants included the Fast Track intervention (*n* = 445; 72.4% male) and high-risk control samples (*n* = 446; 66.4% male). Direct and total effects of random assignment to Fast Track on age 31 outcomes were not significant. However, our analyses showed that Fast Track’s improvements to interpersonal and intrapersonal skills in childhood served as catalysts for better life outcomes at age 31. Higher interpersonal skills led to fewer externalizing, internalizing, and substance use problems, reduced criminality and sexual partners, in addition to increased general health and full-time employment. Improved intrapersonal skills led to greater strength. There were no significant indirect pathways via academic skills. Our findings inform understanding of *how* a childhood preventive intervention can improve adjustment and behaviors into established adulthood.

**Supplementary Information:**

The online version contains supplementary material available at 10.1007/s11121-024-01736-0.

Children with conduct problems are up to three times more likely to be depressed, eight times more likely to be homeless, and 32 times more likely to commit suicide in adulthood, relative to those without conduct problems (Odgers et al., 2007). In addition, a one standard deviation increase in kindergarten conduct problems is associated with a USD $146,279 increase in adult criminal, victim, and medical costs (Goulter et al., 2023). The severity of these problems requires interventions to prevent the pathway from childhood conduct problems to maladjustment in adulthood, and there is strong evidence that early prevention is the key to disrupting this pathway (Conduct Problems Prevention Research Group [CPPRG], 2020; Dodge et al., 2015). The Fast Track intervention is one such early prevention program; it is a comprehensive multicomponent psychosocial intervention designed to prevent the emergence of conduct problems in childhood (CPPRG, 2020). Targeted toward children at risk for conduct problems, numerous evaluations of this intervention have documented improved outcomes across adolescence and early adulthood, including reducing delinquency and depression (CPPRG, 2020; Dodge et al., 2015).

While research has documented Fast Track intervention effects at age 25, its impacts in established adulthood and the mechanisms by which the intervention yields positive effects in established adulthood remain unknown. This study addresses both questions. Specifically, this study examined whether assignment to the Fast Track intervention decreased adverse outcomes at age 31 and how the intervention’s improvements to its participants’ academic, intrapersonal, and intrapersonal skills in childhood contributed to reducing the probability of adverse outcomes at age 31.

## Fast Track Theory and Effects

The CPPRG hypothesized that comprehensive intervention from childhood to adolescence could improve social development and decrease criminality in high-risk children (CPPRG, 1992). Conduct problems emerge early in childhood due to a combination of factors, such as neuropsychological deficiencies (Moffitt, 1993) that are exacerbated by maladaptive parenting behaviors and difficulties in school (Coie & Dodge, 1998). Grounded in ecological and developmental theories emphasizing these many influences on child behavior, the Fast Track project incorporated multiple intervention components during grades one through 10 to disrupt the pathways from conduct problems in childhood to psychopathology in adulthood. Broadly, the elementary school component targeted social, cognitive, and emotional deficits associated with peer victimization, academic difficulties, and oppositional behavior toward parents and teachers. During the middle and high school component, adolescent developmental issues were targeted (e.g., romantic relationships, substance use). The intervention also included home visits for the purpose of increasing parents’ problem-solving and life management skills and parent management training groups to teach behavior management skills and promote the development of positive family-school relationships. The CPPRG predicted that this combination of strategies over time would deter later-in-life problems developing from antisocial behavior in ways that prior, targeted interventions could not. The intervention was evaluated through a randomized controlled trial including 891 children across four U.S. communities and three cohorts (children starting kindergarten in 1991, 1992, and 1993).

The Fast Track intervention has succeeded in improving outcomes from childhood to early adulthood. Specifically, the intervention reduced aggressive behavior and conduct problems in elementary school (CPPRG, 2004). In adolescence, the intervention limited delinquent behaviors in high school (CPPRG, 2007) and juvenile arrests (CPPRG, 2010). Participation in the intervention decreased the probability of suicidal ideation, hazardous drinking, and opioid use in early adulthood (Godwin & CPPRG, 2020). At age 25, intervention participants also had fewer externalizing, internalizing, and substance use problems, less risky sexual behavior, higher well-being, and fewer crime conviction outcomes (Dodge et al., 2015).

Here, we examine the same comprehensive set of outcomes in established adulthood at age 31 previously investigated at age 25 (Dodge et al., 2015). Historically, established adulthood has been overlooked as a critical developmental period, but this life stage is characterized by a distinct set of pressures termed the “career-and-care crunch” (Mehta et al., 2020, p. 436). Other theoretical models emphasize desistance in criminal offending and related behaviors (e.g., externalizing psychopathology, substance use problems) as people move into adulthood (Moffitt, 2010). From an intervention perspective, improved outcomes for Fast Track participants further into adulthood might benefit not just the participants themselves but their romantic partners and children as well. In fact, recent research has confirmed the intergenerational transmission of benefits from the Fast Track intervention at age 34, including effects on family formation (Lansford et al., 2023), healthy family environments (Rothenberg et al., 2023), and reduced health service use in children of Fast Track participants (Rothenberg et al., 2024).

## Fast Track Mechanisms

The present study examined potential mechanisms that account for how Fast Track can improve adult outcomes. Past work has examined three childhood competencies—interpersonal, intrapersonal, and academic—as mechanisms for how Fast Track fosters positive adult adjustment, including reduced criminality, substance use problems, and mental health problems (Sorensen et al., 2016). By analyzing age 31 outcomes through the lens of the intervention’s intended mechanisms of action, the present study helps to explain which childhood competencies contributed to delivering positive outcomes to Fast Track participants in established adulthood.

### Interpersonal Skills

The Fast Track intervention contributed to developing interpersonal skills in childhood (Sorensen et al., 2016). Specifically, the Fast Track intervention contributed to both the promotion of prosocial skills (Dodge et al., 2015; Sorensen et al., 2016) and reductions in antisocial, aggressive behavior that alienated peers (CPPRG, 2020). Components of Fast Track including social skill training groups, peer-pairing, and PATHS all fostered prosocial behavior and friendship (Sorensen et al., 2016). These same components along with parent behavior management training limited aggressive peer relations and externalizing behaviors that alienate peers (Sorensen et al., 2016). As a result, the intervention’s improvement of interpersonal skills decreased the probability of exhibiting behavior of despair in adolescence and young adulthood (Godwin & CPPRG, 2020). Substantial research also confirms that higher childhood interpersonal skills lead to greater overall adult adjustment across multiple outcomes. A childhood intervention designed to improve social skills was associated with increased educational attainment, the probability of employment, the likelihood of marriage, and decreased criminality for young adults (Algan et al., 2022). On the other hand, individuals experiencing social withdrawal in childhood were more likely to develop depression and poor relationships with family and romantic partners (Katz et al., 2011). Poor peer relations draw individuals to criminality too. Associating with deviant peers increased the likelihood for adolescents to commit crimes (Cochran et al., 2017). Combining these two bodies of evidence suggests that participants in Fast Track experience improvements in interpersonal skills (i.e., promotion of prosocial behavior, reduction in antisocial, aggressive behavior) that when carried into adulthood should benefit mental health, education and employment, and romantic and family functioning and reduce criminality due to the wider, deeper, and more prosocial support networks such skills promote (Godwin & CPPRG, 2020; Sorensen et al., 2016).

### Intrapersonal Skills

Previous evaluations of Fast Track reveal that the intervention improves intrapersonal skills by mid-adolescence (Sorensen et al., 2016). Fast Track does so by teaching children to take new perspectives on social situations to reduce their hostile attributions, by teaching children how to use social problem-solving and emotion regulation strategies to maintain control in difficult circumstances and by developing appropriate ways to cope with difficult emotions. In turn, increases in these intrapersonal skill competencies reduce criminality at age 20 (Sorensen et al., 2016). Additionally, prior research also confirms that childhood intrapersonal skills contribute to more positive and fewer adverse adult outcomes. For example, greater social-emotional skills in kindergarten were associated with an improved likelihood of earning a college degree, gaining stable employment, and avoiding an arrest (Jones et al., 2015). Children with higher self-control were less likely to develop substance dependence, depression, anxiety, and aggression, more likely to be in a higher socioeconomic status, and less likely to have been convicted of a crime in adulthood (Moffitt et al., 2011; Robson et al., 2020). In other words, improvements in intrapersonal perspective-taking, problem-solving, and regulation brought about by Fast Track in childhood are critical intrapersonal skills that promote adult functioning across a wide swath of domains, including health, education and employment, and romantic and family functioning (Moffitt et al., 2011; Sorensen et al., 2016).

### Academic Competencies

The Fast Track intervention improved some academic outcomes for participants (Sorensen et al., 2016) in the elementary school years. The intervention’s tutoring component promoted emergent literacy skills. Accordingly, greater academic competence partially explained Fast Track’s influence on lowering mental health service utilization in adolescence and young adulthood (Sorensen et al., 2016). Though no study has investigated whether these Fast Track effects extend further into adulthood, extant research confirms that greater reading readiness in childhood is related to positive outcomes in adulthood. For example, higher reading achievement at age 7 was associated with higher socioeconomic status, academic motivation, and education level (Ritchie & Bates, 2013). Moreover, underachievement in elementary and middle school was associated with a higher probability of developing an adult substance use problem (Fothergill et al., 2008). Finally, lower school performance and engagement increase the risk of criminal offending (Groot & van den Brink, 2010). In sum, Fast Track promotes academic competence that in turn provides adults with opportunities for educational and employment enhancement (Ritchie & Bates, 2013), better mental health, and lower risk for substance use problems and criminality (Fothergill et al., 2008; Groot & van den Brink, 2010).

## Current Study

Using an intent-to-treat design, this study evaluated the impact of intervention assignment on outcomes through age 31 and examined the mechanisms by which these benefits were achieved. We did so by simultaneously examining both the direct and indirect pathways through which Fast Track conveyed its beneficial effects at age 31 (see Supplementary Figure S1). Given the beneficial effects of Fast Track on the same outcomes examined here at age 25 (Dodge et al., 2015), we hypothesized that the Fast Track intervention continued to prevent adverse outcomes and improve overall adjustment at age 31. Specifically, intervention participants would have lower substance use problems, lower risky sexual behavior, greater happiness, and lower violent and substance crime conviction outcomes. Moreover, we tested the pathways by which Fast Track might convey these effects. Given that higher levels of interpersonal, intrapersonal, and academic competencies contribute to positive adult outcomes, we expected to find that Fast Track’s impacts in these areas were the pathways by which Fast Track positively affected age 31 outcomes.

## Method

### Participants and Procedure

Between 1991 and 1993, 55 high-risk schools were selected for Fast Track participation based on crime and poverty rates of their communities across four sites (Durham, NC; Nashville, TN; Seattle, WA; and rural Pennsylvania; CPPRG, 2020). Characteristics of the schools varied across sites: Durham, 90% ethnic minority, 80% of students qualifying for free/reduced-priced lunch; Nashville, 54% ethnic minority, 78% of students qualifying for free/reduced-priced lunch; Seattle, 52% ethnic minority, 45% of students qualifying for free/reduced-priced lunch; and rural Pennsylvania, 1% ethnic minority, 39% of students qualifying for free/reduced-priced lunch. Within each site, clusters of schools were randomly assigned to intervention and control conditions. Children were selected for inclusion into the sample based on teacher and parent reports of behavior problems. Teachers screened 9594 kindergarteners for classroom conduct problems using the Teacher Observation of Classroom Adaptation-Revised authority acceptance score (Werthamer-Larsson et al., 1991). Those children scoring in the top 40% within cohort and site were then solicited for the next stage of screening for home behavior problems by the parents, using 22 items from the Child Behavior Checklist (Achenbach, 1991) and similar scales to capture behavior problems at home; 91% agreed (*n* = 3274). Teacher and parent screening scores were standardized within site and summed to yield a total severity-of-risk screen score. Children were selected for inclusion into the high-risk sample based on this screen score, moving from the highest score downward until desired sample sizes were reached within sites, cohorts, and groups. This multi-stage screening procedure resulted in an intervention (*n* = 445; 72.4% male; 53.0% Black) and high-risk control (*n* = 446; 66.4% male; 48.4% Black) sample. Legal guardians provided consent and participants assented to procedures. Participants were provided monetary compensation for their time. All procedures were approved by the Institutional Review Boards of participating universities.

During the elementary school phase of the intervention (grades 1–5), all intervention families were offered parent management training with home visiting, academic tutoring, and child social skills training. Parent and child group interventions were conducted during a 2-h enrichment program. These sessions included social skill training 'friendship groups' (Bierman et al., 2017), parent management training groups for parents, and guided parent–child interaction sessions (McMahon et al., 1996). Paraprofessionals also provided tutoring, as well as peer-pairing sessions to improve friendships with classmates. In addition, a universal intervention (a modified, grade-level version of the PATHS curriculum; Greenberg et al., 2011) was provided to the classrooms in intervention schools through the elementary school years to promote social and emotional competence. The universal intervention included weekly teacher consultation for lessons and classroom behavior management (Bierman et al., 2017).

During the middle and early high school phase (grades 6–10), there were three standard prevention activities offered to all FT intervention children: the middle school transition program, parent and youth groups on adolescent topics, and youth forums. Adolescent developmental issues were addressed with four meetings for parents and youth during grade 6. Parent groups focused on issues such as positive involvement and monitoring, and youth groups focused on issues such as coping with peer pressure. Parents and youth met together in groups to address romantic relationships and sex education, substance use, and vocational goal setting. In grades 7 and 8, eight Youth Forums based on Oyserman’s (2000) program were held with youth in small groups to address vocational opportunities, budgeting and life skills, job interview skills, and summer employment opportunities. In grades 7–10, individualized intervention plans were developed and implemented with each youth, based on regular assessments of risk and protective factors, conducted three times each year. Further information regarding the intervention components is provided in Supplementary Table S1.

This study used data from the age 31 follow-up survey (*n* = 657; 66.4% male; 51.8% Black; 50.4% assigned to intervention). As detailed in the Supplementary Materials, comparing those with age 31 data to those without across 25 baseline variables (capturing intervention assignment, demographics, child behaviors, and parenting behaviors), only 2 significant differences emerged (sex and site). We concluded that attrition (26%) did not disrupt the representativeness of the sample. To control for differences and improve estimation precision and power, these 25 variables were included in the models estimated with full information maximum likelihood (in line with past Fast Track analyses such as Dodge et al., 2015 and Godwin & CPPRG, 2020).

### Measures

All measures are described in detail in the Supplementary Materials (including variable construction and reliability statistics). Descriptive statistics are found in Table 1.

**Table 1:** Descriptive statistics by intervention status

	**Intervention**		**Control**	
	***N***	***M* (SD)**	***N***	***M* (SD)**
**Age 31 outcomes**				
**Any externalizing, internalizing, or substance use problem**^**a**^	331	0.44	322	0.43
**Externalizing problems *T*-score**	331	52.04 (12.69)	322	51.84 (11.59)
Antisocial Personality DSM Clinical Range^a^	331	0.12	322	0.09
ADHD Problems DSM Clinical Range^a^	331	0.06	322	0.06
**Internalizing problems *T*-score**	331	52.73 (13.9)	322	53.82 (13.46)
Avoidant Personality DSM Clinical Range^a^	331	0.06	322	0.10
Somatic DSM Clinical Range^a^	331	0.13	323	0.12
Anxiety DSM Clinical Range^a^	331	0.06	322	0.07
Depression DSM Clinical Range^a^	331	0.11	322	0.09
**Any problematic substance use**^**a**^	325	0.28	322	0.26
Binge drinking problem^a^	316	0.08	316	0.09
Heavy cannabis use^a^	321	0.14	317	0.13
Serious substance use^a^	330	0.09	323	0.12
**Strength**	331	16.25 (4.01)	322	16.28 (3.83)
**General health index**	331	0.74 (0.25)	323	0.73 (0.25)
**Education and employment**				
High school diploma/GED^a^	326	0.84	317	0.79
Currently full-time employed^a^	321	0.45	315	0.50
**Romantic relationships**				
Number of sexual partners over a lifetime	321	3.79 (1.99)	316	3.81 (2.00)
Risky sexual behavior in the past 12 months	301	12.16 (41.85)	304	10.82 (23.28)
Any intimate partner violence^a^	286	0.16	277	0.15
**Parenting**				
Coercive parenting	190	0.13 (0.39)	187	0.13 (0.28)
Spanking scale	190	0.61 (0.92)	187	0.71 (0.96)
**Severity-weighted conviction indices**				
All crime^b^	423		427	
0		0.42		0.43
1		0.01		0.00
2		0.06		0.06
3		0.04		0.04
4		0.02		0.02
5		0.03		0.03
6 +		0.43		0.42
Drug crime^b^	423		427	
0		0.67		0.74
1		0.10		0.05
2		0.09		0.05
3		0.05		0.02
4		0.02		0.04
5		0.02		0.02
6 +		0.05		0.08
Violent crime^b^	423		427	
0		0.60		0.60
1		0.10		0.11
2		0.08		0.07
3		0.05		0.06
4		0.06		0.03
5		0.02		0.02
6 +		0.10		0.12
Property & public order crime^b^	423		427	
0		0.56		0.56
1		0.01		0.01
2		0.07		0.10
3		0.03		0.02
4		0.06		0.06
5		0.03		0.03
6 +		0.24		0.23
**Childhood/adolescent competencies**				
Interpersonal skills	428	0.03 (0.87)	414	-0.38 (0.96)
Intrapersonal skills	430	0.12 (0.97)	425	-0.21 (1.01)
Academic skills	431	0.00 (0.92)	429	-0.18 (1.15)

^*^p < .05

^a^Outcome is dichotomous and proportions are reported

^b^Outcome is a count variable and proportions are reported for selected values

#### Adult Outcomes at Age 31

##### Externalizing and Internalizing Psychopathology

Self-reports of externalizing and internalizing problems were assessed using the 132-item Adult Self-Report (ASR, Achenbach, 1997). Indicators were included for endorsing criteria for clinical range of DSM-IV diagnoses for antisocial personality disorder (ASPD), attention deficit hyperactivity disorder (ADHD), avoidant personality, somatic disorder, anxiety, and depression.

##### Substance Use

Self-reports of substance use (including regular binge drinking, heavy cannabis use, other substance use, and an 'any substance use problem' indicator) were assessed with the 57-item Tobacco, Alcohol, and Drugs Survey-Version 3 adapted from the National Longitudinal Survey of Youth (Bureau of Labor Statistics, 2002).

##### Any Externalizing, Internalizing, or Substance Use Problem

This indicator was scored as 1 if criteria for any of the following problems were present, or 0 otherwise: ASPD, ADHD, avoidant personality, somatic disorder, anxiety, depression, binge drinking, heavy cannabis use, or other substance use.

##### Strength

To capture elements of positive psychology (Seligman, 2011) including happiness, engagement, relationships, meaning, and accomplishment, a strength score was created by summing across 11 ASR items including being happy, making good use of opportunities, enjoying being with others, standing up for rights, and working up to ability.

##### General Health Index

Four items from the Short-Form Health Survey (Ware & Sherbourne, 1992) were used to create a general health index with higher scores indicating better overall health status, absence of chronic conditions, less bodily pain, and fewer physical health issues.

##### Education and Employment

Two dichotomous scores indicating whether (a) the participant graduated from high school or received a GED and (b) was currently employed full-time were created from a measure adapted from the National Longitudinal Survey (Howe & Frazis, 1992).

##### Sexual Behavior

The 37-item Overview of Sexual Experiences (Capaldi et al., 2002) assessed self-reported risky sexual behavior.

##### Any Intimate Partner Violence

Self-reports of partner violence were measured with the 47-item General Violence Questionnaire (Holtzworth-Munroe et al., 2000).

##### Parenting

Participants with children completed the Conflict Tactics Scale (Straus, 1979) which was used to construct two scales summarizing the use of coercive parenting and spanking.

##### Criminal Offenses

Administrative court records were collected using national databases (based on full name, birthdate, and social security number) that included all arrests, adjudications, diversions, and magistrate appearances. We limited offenses to convictions for violent, substance, and property or public order crimes. Severity-weighted indices were created by multiplying frequencies with severity across all lifetime adult convictions (CPPRG, 2010).

#### Mechanisms of Action: Elementary and Middle School Skills

These scores were designed as indices to aggregate the skills intervention youth acquired from 1st through 8th grade in each of these domains (Godwin & CPPRG, 2020). Detailed descriptions and scoring for all mechanisms of action can be found in Supplementary Table S3.

##### Interpersonal Skills

Interpersonal skills reflect the child’s actual behavior toward others and in interactions with others. This was a composite that included 15 scores comprising prosocial behaviors including positive child behavior, positive peer relations, and antisocial behaviors including oppositional and aggressive behavior (reverse coded) across grades 1 through 8 (Godwin & CPPRG, 2020).

##### Intrapersonal Skills

Intrapersonal skills reflect internal processes that the child uses to interpret social interactions and plan their responses to these interactions. This was a composite measure that included seven scores including competent social problem-solving, emotional recognition, and hostile attributions (reverse coded) across grades 1 through 5 (Godwin & CPPRG, 2020).

##### Academic Competencies

Academic skills were a composite measure that included four scores including diagnostic reading and special education services (reversed) across grades 1 through 3 (Godwin & CPPRG, 2020).

### Analytic Approach

Traditionally, assessments of Fast Track have evaluated the total effect of assignment to intervention capturing direct and indirect pathways by which intervention influences the outcome—estimating the total effect on a single outcome using an indicator for assignment to intervention (Dodge et al., 2015). Secondary analyses have explored whether significant total intervention effects were mediated by the intervention’s impact on childhood and adolescent competencies targeted by the intervention—interpersonal, intrapersonal, and academic skills (Sorensen et al., 2016). However, for investigations like the current one, which is focused specifically on Fast Track’s mechanisms of action, quantitative psychologists have found that this classic causal step approach of establishing a statistically significant total effect before examining mediational effects is underpowered and not necessary or recommended (Hayes, 2009; MacKinnon et al., 2000; Shrout & Bolger, 2002). Instead, when exploring questions about mediating mechanisms of action, quantitative psychologists recommend more rigorous approaches such as bootstrapping that (a) simultaneously but separately model the “direct” and “indirect” components of a total intervention effect and (b) directly evaluate the mediating “indirect” path (MacKinnon et al., 2004). We adopted this recommended approach in the current study. However, to replicate the evaluation of the Fast Track intervention most directly on age 25 outcomes (Dodge et al., 2015), we provide the results from analyses estimating the traditional total intervention effects on age 31 outcomes in the Supplementary Materials.

To simultaneously estimate the direct and indirect effects of assignment to intervention on each age 31 outcome, we estimated a single four-outcome path model including the age 31 outcome, interpersonal skills, intrapersonal skills, and academic competencies. Each competency was modeled as a function of intervention status, site, cohort, race, sex, and 20 pre-intervention covariates that have historically been included in FT intervention studies (Dodge et al., 2015). The age 31 outcome was estimated as a function of the same predictors plus the three competency scores with the coefficient on intervention status capturing the direct effect of intervention. The indirect pathways from assignment to intervention to the age 31 outcome through each type of competency are estimated using the product of coefficients method with 1000 bootstrapped samples (Sim et al., 2022) to obtain 95% confidence intervals of the indirect path effect (MacKinnon et al., 2004). For example, the indirect pathway from intervention to the age 31 outcome through interpersonal skills was the product of the intervention effect on interpersonal skills and the effect of interpersonal skills on the age 31 outcome. All analyses were conducted in *Mplus* version 8.8 (Muth´en & Muth´en, 2021) with Monte Carlo integration, standard errors clustered by kindergarten school to account for the randomization process, and full information maximum likelihood to provide full-sample estimates that accommodate all observations regardless of whether missing data occur for certain variables (Rubin & Little, 2002).

For dichotomous outcomes, logistic regression models were estimated, and results were reported as odds ratios (ORs) and number needed to treat (NNT, Mendes et al., 2017). For the severity-weighted conviction indices, zero-inflated Poisson models were estimated to account for the high proportion of participants who were never convicted of an adult offense (index = 0). These models estimated the impact of assignment to intervention on (1) the probability of *never* being convicted of an adult crime (reported as ORs) and (2) the expected severity-weighted index among those who were convicted of a crime (reported as incidence rate ratios [IRR]). Consequently, the indirect effects were also estimated for the probability of *never* being convicted as well as the estimated index among those who were convicted of at least one offense. Given the current study’s control for Type I error via inclusion of numerous covariates and use of bootstrap estimation, nominal *p* values were reported instead of adjusted for multiple comparisons.

## Results

### Direct Effects of Intervention

As seen in the second column of Table 2, the direct effect of assignment to intervention was significantly associated with two of the 28 age 31 outcomes. Unexpectedly, the odds of being employed full-time were 45% lower for intervention participants relative to control participants (OR = 0.55). Also unexpectedly, the odds of *never* being convicted of a substance-related crime by age 31 were 46% lower for intervention participants relative to control participants (OR = 0.54). Similar findings emerged when total effects were examined (see Supplementary Materials).

**Table 2:** Direct and indirect intervention effects

	Direct	Indirect intervention effects through		
	Intervention	Interpersonal skills	Intrapersonal skills	Academic skills
Any externalizing, internalizing, or substance use problem^a^	1.19 (0.84, 1.69)	0.89 (0.80, 0.97)*	0.98 (0.89, 1.05)	1.01 (0.99, 1.05)
Externalizing problems *T*-score	1.38 (− 0.54, 2.97)	− **0.91 (**− **1.57,** − 0.36)*	− 0.18 (− 0.58, 0.21)	− 0.05 (− 0.24, 0.07)
Antisocial Personality DSM Clinical Range^a^	1.47 (0.75, 2.92)	0.89 (0.71, 1.03)	1.08 (0.97, 1.29)	0.99 (0.94, 1.03)
ADHD Problems DSM Clinical Range^a^	1.45 (0.55, 4.18)	0.86 (0.61, 1.09)	0.94 (0.77, 1.18)	0.96 (0.87, 1.02)
Internalizing problems *T*-score	0.26 (− 1.83, 2.08)	− **0.73 (**− **1.43,** − 0.15)*	− 0.28 (− 0.78, 0.14)	− 0.08 (− 0.32, 0.08)
Avoidant Personality DSM Clinical Range^a^	0.71 (0.32, 1.42)	0.98 (0.80, 1.16)	0.99 (0.86, 1.16)	0.98 (0.91, 1.02)
Somatic DSM Clinical Range^a^	1.29 (0.77, 2.29)	0.87 (0.75, 0.98)*	0.97 (0.87, 1.06)	0.98 (0.93, 1.02)
Anxiety DSM Clinical Range^a^	0.92 (0.34, 2.24)	0.98 (0.77, 1.28)	0.92 (0.77, 1.09)	0.98 (0.92, 1.05)
Depression DSM Clinical Range^a^	1.21 (0.54, 2.60)	0.92 (0.73, 1.12)	0.96 (0.82, 1.11)	1.00 (0.94, 1.06)
Any problematic substance use^a^	1.12 (0.76, 1.75)	0.89 (0.80, 0.98)*	1.02 (0.93, 1.13)	1.02 (0.98, 1.07)
Binge drinking problem^a^	0.86 (0.36, 2.02)	0.94 (0.78, 1.13)	1.03 (0.86, 1.24)	1.02 (0.97, 1.11)
Heavy cannabis use^a^	1.35 (0.83, 2.37)	0.84 (0.70, 0.94)*	0.96 (0.84, 1.07)	1.03 (0.99, 1.11)
Serious substance use^a^	0.74 (0.33, 1.35)	0.89 (0.74, 1.03)	1.09 (0.97, 1.34)	1.01 (0.96, 1.11)
Strength	− 0.33 (− 1.00, 0.29)	0.09 (− 0.06, 0.24)	0.15 (0.01, 0.33)*	0.00 (− 0.04, 0.05)
General health index	− 0.02 (− 0.06, 0.01)	0.01 (0.00, 0.02)**	0.00 (0.00, 0.01)	0.00 (0.00, 0.00)
Education and employment				
High school diploma/GED^a^	0.99 (0.59, 1.63)	1.08 (0.98, 1.23)	1.07 (0.97, 1.19)	1.03 (0.99, 1.08)
Currently full-time employed^a^	0.55 (0.36, 0.79)*	1.13 (1.04, 1.27)*	1.01 (0.93, 1.08)	1.05 (0.99, 1.12)
Romantic relationships				
Number of sexual partners over a lifetime	− 0.08 (− 0.40, 0.26)	− **0.09 (**− **0.17,** − 0.02)*	0.06 (− 0.01, 0.12)	0.02 (− 0.01, 0.05)
Risky sexual behavior in the past 12 months	0.87 (− 3.20, 6.38)	− 0.51 (− 1.94, 0.70)	0.35 (− 0.83, 1.54)	0.17 (− 0.12, 0.62)
Any intimate partner violence^a^	1.21 (0.67, 2.57)	0.96 (0.84, 1.09)	0.97 (0.84, 1.11)	1.03 (0.99, 1.10)
Parenting				
Coercive parenting	0.03 (− 0.05, 0.12)	− 0.01 (− 0.03, 0.01)	0.01 (− 0.01, 0.03)	0.00 (0.00, 0.01)
Spanking scale	− 0.03 (− 0.23, 0.18)	0.00 (− 0.05, 0.04)	0.01 (− 0.03, 0.05)	0.01 (− 0.01, 0.03)
Severity-weighted conviction indices^b^				
All types of crime				
Probability of no convictions	0.80 (0.54, 1.13)	1.17 (1.10, 1.29)*	1.00 (0.93, 1.07)	1.00 (0.98, 1.03)
Expected index among those convicted	1.02 (0.84, 1.23)	0.92 (0.87, 0.96)*	0.99 (0.95, 1.02)	1.00 (0.99, 1.01)
Substance crime				
Probability of no convictions	0.54 (0.28, 0.82)*	1.18 (1.07, 1.37)*	1.03 (0.93, 1.13)	0.99 (0.96, 1.02)
Expected index among those convicted	0.80 (0.57, 1.07)	0.97 (0.90, 1.02)	0.99 (0.93, 1.04)	1.00 (0.97, 1.02)
Violent crime				
Probability of no convictions	0.74 (0.45, 1.06)	1.21 (1.11, 1.38)*	1.05 (0.98, 1.16)	1.00 (0.98, 1.04)
Expected index among those convicted	0.82 (0.61, 1.17)	0.96 (0.89, 1.02)	1.01 (0.96, 1.09)	0.99 (0.98, 1.01)
Property/public order crime				
Probability of no convictions	0.79 (0.55, 1.06)	1.19 (1.09, 1.30)*	1.02 (0.96, 1.09)	1.01 (0.99, 1.04)
Expected index among those convicted	1.05 (0.74, 1.36)	0.93 (0.88, 1.09)	0.98 (0.93, 1.02)	1.01 (0.99, 1.03)

^*^p < .05

^a^Outcome is dichotomous and the intervention effect was modeled using a logistic regression and presented as an OR

^b^Outcome is a count variable, and the intervention effect was modeled using a zero-inflated Poisson regression with the impact of intervention on the probability of a structural zero presented as an OR and the intervention effect on the expected count among those with a non-zero value presented as an IRR. Complete set of results is available upon request. Independent tests were conducted for each outcome

### Indirect Effects of Intervention Through Interpersonal Skills

As seen in the third column of Table 2, there was evidence of significant indirect intervention effects through interpersonal skills across a wide range of adult outcomes. The indirect effect of intervention through interpersonal skills was associated with an 11% decrease in the odds of experiencing any externalizing, internalizing, or substance use problem at age 31 (OR = 0.89, NNT = 35). Similarly, this indirect intervention pathway was associated with lower externalizing and internalizing problem *T*-scores (*b* =  − 0.91 and − 0.73, respectively) and an 11% decrease in the odds of any problematic substance use (including regular binge drinking, heavy cannabis use, or any other substance use, OR = 0.89, NNT = 46). In addition, the indirect effect of intervention through interpersonal skills was associated with a 13% decrease in the odds of meeting the clinical criteria for somatic disorder at age 31 (OR = 0.87, NNT = 72) and a 16% decrease in the odds of heavy cannabis use (OR = 0.84, NNT = 54).

In addition to indirect pathways to problematic adjustment and substance use, there was evidence of significant beneficial indirect intervention effects on positive adjustment through interpersonal skills acquired in childhood and early adolescence. The indirect effect of intervention through interpersonal skills was associated with higher overall general health (*b* = 0.01). In addition, this indirect intervention pathway was associated with a 13% increase in the odds of full-time employment at age 31 (OR = 1.13; NNT = 33) and a lower estimated number of lifetime sexual partners (*b* =  − 0.09).

Finally, there was strong evidence that the intervention indirectly impacted criminality through interpersonal skills. The indirect effect of intervention through interpersonal skills was associated with a 17% increase in the odds of *never* being convicted of any adult crime by age 31 (OR = 1.17, NNT = 26) and an 8% decrease in the expected severity-weighted index of convictions among those convicted by age 31 (IRR = 0.92). Similarly, the indirect effect of intervention through interpersonal skills was associated with an increase in the odds of *never* being convicted of specific types of crimes: substance-related (18% increase, NNT = 33), violent (21% increase, NNT = 22), and property/public order (19% increase, NNT = 24) crimes.

### Indirect Effects of Intervention Through Intrapersonal Skills

As seen in the fourth column of Table 2, there was no evidence of significant indirect effects through intrapersonal skills on problematic adjustment and substance use; however, the indirect effect of intervention through intrapersonal skills was associated with higher strength (*b* = 0.15).

### Indirect Effects of Intervention Through Academic Skills

As seen in the fifth column of Table 2, there was no evidence of significant indirect intervention effects on age 31 outcomes through academic skills acquired in childhood.

## Discussion

We examined whether the Fast Track intervention continued to improve outcomes in established adulthood—15 years after the intervention ended. Our study hypotheses that Fast Track participation would improve adult outcomes by strengthening childhood interpersonal, intrapersonal, and academic skills were partially supported. When Fast Track improves interpersonal skills and intrapersonal skills in elementary school, those skill improvements subsequently lead to improvements in several age 31 outcomes. The Fast Track intervention improved interpersonal skills, leading to fewer externalizing, internalizing, and substance use problems, reduced criminality (including substance, violent, and property crimes) and number of sexual partners, and increased general health and full-time employment. When the Fast Track intervention improved intrapersonal skills, those improvements led to better strength. However, it is also important to note that, on average, there were few beneficial direct or total effects of assignment to the Fast Track intervention on age 31 outcomes. That means that Fast Track’s beneficial effects on age 31 outcomes depend upon its ability to change interpersonal and intrapersonal skills in elementary school. When Fast Track was able to do so, better age 31 outcomes resulted. Otherwise, such beneficial outcomes are largely absent.

### Identifying Mechanisms of Action that Explain Long-Term Effects of Fast Track

Development of intrapersonal skills and especially interpersonal skills in childhood were pathways through which the Fast Track intervention improved adult outcomes. We suspect Fast Track’s development of these skills contributed to improved outcomes in adulthood because of their ability to help avoid antisocial peers, the developmental timing when these skills were learned, and their ability to provide a positive lens through which one can interpret the world.

Building interpersonal skills through the intervention could have helped participants avoid antisocial peers that can act as a bridge to criminality, substance use problems, and other adult difficulties. Deviant peer relationships can predict criminality because deviant peers can make criminality more appealing and accessible (Costello & Hope, 2016). Similarly, peer influence is a primary reason for engaging in substance use during adolescence and serves as a linchpin in developmental pathways from early childhood externalizing and internalizing behaviors to adult substance use problems and criminality (Hussong et al., 2018). Social skill training through Fast Track promoted participants’ prosocial behavior, making them more likely to associate with positive peers and less likely to associate with deviant peers. Avoiding antisocial peers may be one way Fast Track shifts individuals off long-run trajectories toward adverse adult outcomes.

In addition, interpersonal skills that Fast Track improved could be a critical mechanism of action because the intervention improved those skills at important points in the participants’ lifetimes. Externalizing and internalizing problems and substance use start to emerge in middle childhood and adolescence, and each has been strongly linked to interpersonal skill deficits (Fanti & Henrich, 2010; Hussong et al., 2018). Fast Track built interpersonal skills through multiple social skills and social-ecological interventions during middle childhood to protect against stronger peer influence in adolescence to substance use and risky behaviors. Therefore, Fast Track’s building of interpersonal skill competencies may have been delivered at the period in development needed to prevent individuals from experiencing interpersonal skill deficits as entrée points to lifelong trajectories toward criminality, substance use, and other difficulties (CPPRG, 2020).

Intrapersonal skills developed through the Fast Track intervention acted as a mechanism for greater strength scores (including being happy, making good use of opportunities, enjoying being with others, standing up for rights, and working up to ability) at age 31. Fast Track promoted adaptive intrapersonal skills such as higher emotion regulation, less hostile attribution bias, and effective coping (Sorensen et al., 2016). Development of these intrapersonal skills allows individuals to both recognize the world as a less hostile, frightening place and to respond with appropriate perspective and cognitive reappraisal when things go wrong (Compas et al., 2014; Sorensen et al., 2016). Therefore, collectively, these strategies allow individuals to see themselves and their world more positively (Compas et al., 2014; Sorensen et al., 2016). In other words, intrapersonal skills can guide perceptions of an individual’s life, and those perceptions can shape an individual’s strength. Fast Track contributed to participants having more optimistic perceptions of their world by promoting intrapersonal skills in childhood.

Notably, the intervention delivered few benefits at age 31 through academic skills. This may be because academic skills were measured at more developmentally distal time points than interpersonal skills. Specifically, this study measured Fast Track’s effects on interpersonal skills through eighth grade, while measures of academic gains ended in third grade. The intervention implemented systematic tutoring in the first 2 years of the program, but later academic supports were less intensive. Since academic skills were measured earlier in childhood and academic-focused interventions declined, it is more conceivable that the effects may not be as long-lasting.

### Nonsignificant and Unexpected Intervention Effects at Age 31

For some externalizing problems (e.g., Antisocial Personality DSM score, ADHD Problems DSM score), internalizing problems (e.g., Anxiety DSM and Depression DSM scores), and substance use behaviors (e.g., binge drinking and serious substance use) that were serious enough to be considered in the “clinically elevated” range, the Fast Track intervention did not have any direct or indirect effects. We suspect Fast Track may not have affected these outcomes because clinically elevated externalizing, internalizing, and substance use problems tend to peak in early adulthood before declining in established adulthood (Hussong et al., 2018; Moffitt, 2010; Solmi et al., 2022). This normative developmental trend of decreasing prevalence could lead control group participants to “catch up” to Fast Track participants in participants’ 30 s as these problems naturally desist. We see this trend in our own data. At age 25, 59% of participants in the Fast Track intervention group and 69% of participants in the Fast Track control group reported experiencing any externalizing, internalizing, or substance use problem (Dodge et al., 2015). By age 31, those proportions had declined to 44% in the intervention group and 43% in the control group, respectively (see Table 1).

In addition to these nonsignificant effects, the Fast Track intervention group was unexpectedly less likely to never have been convicted of a substance crime and less likely to be employed full-time regardless of whether total effects (Supplementary Table S4) or direct effects (Table 2; “Direct” column) were examined. Examining the distribution of substance crimes might explain this effect. As seen in the severity-weighted conviction indices substance crime scores in Table 1, 24% of the Fast Track intervention group had a score of 1–3 on substance crime, compared to only 12% of the control group. However, as substance crimes got more severe, this trend flipped. Fewer in the Fast Track intervention group had a score of 4–6 on substance crime (9%), whereas 14% of the control group did. Therefore, although it is true that the Fast Track intervention group was less likely to have avoided a substance crime conviction than the control group, the Fast Track control group was more likely to commit substance crimes that were more frequent and severe in nature.

Digging deeper into the employment data also offers an alternative explanation for the finding that the Fast Track group was less likely to be employed full-time. Specifically, whereas fewer Fast Track intervention participants reported full-time employment compared to the control group, the exact same proportion of participants (67%) in the Fast Track intervention and control groups reported at least some employment. Moreover, participants in the Fast Track intervention group had children at significantly higher rates than participants in the control group (Lansford et al., 2023). Therefore, we hypothesize that participants in the Fast Track group had lower levels of full-time employment because they were more likely to work part-time outside of the home and spend more time taking care of their children and households.

### Limitations and Future Directions

This study has numerous strengths, including its use of a randomized controlled trial, comprehensive multicomponent intervention across multiple sites with urban/rural and racial diversity, and long-term longitudinal follow-up into established adulthood—an understudied developmental period. However, the current study has limitations that are worth noting. Primarily, this research cannot identify the individual intervention components that affected adulthood because the intervention group received all parts of the intervention. In addition, this study only tested three mechanisms—academic, intrapersonal, and interpersonal skills in childhood—as pathways through which Fast Track impacted adult outcomes. The intervention could have affected adult outcomes through other pathways, such as changing participant stress neurobiology or improving executive functioning. Given the focus of the Fast Track intervention on family factors, parenting behaviors represent another mechanism through which the intervention may impact adult outcomes. Moreover, the intervention’s impact on academic, interpersonal, and interpersonal skills in childhood could have affected adult outcomes through more proximal mediators in early adulthood, and future work could investigate this ongoing cascade of events that Fast Track might have set in motion. Further research into Fast Track’s mechanisms of action could create a complete picture of how the intervention impacts adult outcomes.

### Clinical Applications

Our work shows how the Fast Track intervention can be used in clinical applications as a multicomponent preventive intervention that not only identifies but also ameliorates risk and protective factors that serve to launch children onto trajectories of criminality and antisocial behavior impacting adult outcomes. The multilevel comprehensive nature of the intervention, which reached children at home and in school across 10 years, might contribute to its effectiveness in achieving longer-lasting effects than interventions that are deployed in only a single environment for a limited time. Specifically, incorporating social-emotional learning into classrooms, as Fast Track did, could act as a cost-effective policy to encourage prosocial childhood behavior and avoid detrimental adult outcomes. Additionally, the current study and Dr. McMahon’s work in general go beyond identifying risk and protective factors. This study revealed risk and protective factors that might be changed by the Fast Track intervention and showed how those factors serve as mechanisms of action to improve outcomes across the life course. Moreover, these mechanistic pathways had effects on adult functioning that were modest but notable. Number needed to treat analyses revealed that across significant mediational pathways, 24–72 participants had to traverse these pathways for one participant’s negative adult outcomes to be prevented. As a point of comparison, the median estimated number of people who needed to treat with the COVID-19 vaccine to prevent one hospitalization was 205 (Adams et al., 2023). So, Fast Track’s impacts on age 31 adult outcomes could be conceptualized as a very effective mental health vaccine. Relatedly, this investigation identified not just that Fast Track was effective but why and when it was effective. Identifying the active ingredients in multicomponent interventions that change outcomes across the life course is a critical next frontier in prevention research.

### Conclusion

The Fast Track intervention continued to improve life outcomes for its participants many years after the intervention ended when it improved childhood and adolescent skills. Specifically, our work finds that childhood gains in interpersonal skills and intrapersonal skills due to the Fast Track intervention were a pathway to lower criminality, fewer externalizing and internalizing problems, reduced excessive substance use, and better general health. Multicomponent, comprehensive programs directed at childhood skills can achieve benefits well into adulthood.

## Supplementary Information

Below is the link to the electronic supplementary material.

Supplementary file1 (DOCX 454 KB)

## Data Availability

Data is available upon request to the second author.
