# Supplementary material for: Fast Track Intervention Effects and Mechanisms of Action Through Established Adulthood
Source: Prev Sci. 2024 Oct 11;26(4):667–80. doi: 10.1007/s11121-024-01736-0 (PMC12034041; doi:10.1007/s11121-024-01736-0)
Supplement: Supplementary file 1 — Supplementary file1 (DOCX 454 KB) [file 11121_2024_1736_MOESM1_ESM.docx]

**Fast Track Intervention Effects and Mechanisms of Action Through Established Adulthood Supplementary Materials**

**Missing Data Analyses**

At age 31, 657 of the original 891 Fast Track participants completed the follow-up survey (74%). As seen in Supplementary Table S2, comparing those with age 31 data to those without data across 20 pre-intervention variables (capturing child behaviors and attributes as well as parenting behaviors of the participants’ parents) and 5 demographic characteristics including intervention status revealed only 2 statistically significant differences for sex and site. Attritions was higher for male participants (compared to females) and for participants in Nashville and Seattle (relative to participants in Durham and rural Pennsylvania). Given that only 2 of the 25 differences were significant, we concluded that attrition did not disrupt the representativeness of the sample. However, to control for any existing differences and improve precision in calculating the impact of intervention, these 25 variables were included in the statistical models.

**Measures**

**Adult Outcomes at age 31** (see Dodge et al., 2015 for further details.)

***Externalizing and Internalizing Psychopathology.*** Self-reports of internalizing and externalizing problems were assessed with the 132-item Adult Self-Report (Achenbach, 1997). The externalizing broadband *T*-score ( = 0.93) was comprised of items from the delinquent and aggressive behavior problem narrowband scales, and the internalizing broadband *T*-score ( = 0.94) was comprised of items from the anxious/depressed, withdrawn, and somatic problem narrow-band scales. Items were scored on a 3-point scale (0 ‘*not true*’, 1 ‘*somewhat or sometimes true*’, 2 ‘*very or often true*’). This measure also assessed psychiatric symptoms for antisocial personality disorder (ASPD, = 0.88), attention deficit hyperactivity disorder (ADHD, = 0.86), avoidant personality ( = 0.79), somatic problems ( = 0.82), anxiety ( = 0.78), and depression ( = 0.88). From the sum scores, indicators for meeting DSM-IV criteria for clinical diagnoses were calculated (1 ‘*yes*’, 0 ‘*no*’).

***Substance use*.** Self-reports of substance use were assessed with the 57-item Tobacco, Alcohol, and Drugs Survey-Version 3 adapted from the National Longitudinal Survey of Youth (Bureau of Labor Statistics, US Department of Labor, 2002). The present study included three dichotomous indicators: regular binge drinking (defined as 5 or more drinks on 1 or more occasion in the last month and 5 or more drinks on 12 or more occasions in the last year); heavy cannabis use (defined as 27 or more days of use in the past month); and other substance use (defined as use of cocaine, crack, inhalants, heroin, LSD, phencyclidine, ecstasy, mushrooms, speed, or other pills not prescribed by a physician in the past month). In addition, an ‘any substance use problem’ indicator was scored 1 if any of the substance use problems were met, 0 otherwise.

***Any Externalizing, Internalizing, or Substance Use Problem.*** This indicator was scored as 1 if criteria for any of the following problems were present, or 0 otherwise: anxiety, depression, avoidant personality, somatic problems, ASPD, ADHD, binge drinking, heavy cannabis use, or other substance use.

***General Health Index.*** Participants completed a 20-item health measure adapted from the Short-Form Health Survey (Ware & Sherbourne, 1992). A general health index that comprised a mean score across items capturing overall health status, presence of chronic conditions, magnitude of bodily pain, and presence of physical health issues that impede work ( = 0.71).

***Strength.*** To capture elements of positive psychology (Seligman, 2011) including happiness, engagement, relationships, meaning, and accomplishment, a strength score was created by summing across 11 ASR items. Items included: being a happy person, making good use of opportunities, trying new things, meeting responsibilities to family, enjoying being with others, being fair to others, being honest, standing up for rights, helping others, working up to ability, and doing things better than others ( = 0.71).

***Education and employment.*** Two dichotomous scores indicating whether (a) the participant graduated from high school or received a GED and (b) was currently employed full-time were created from a measure adapted from the National Longitudinal Survey (Howe & Frazis, 1992).

***Sexual behavior****.* The 37-item Overview of Sexual Experiences (Capaldi et al., 2002) assessed self-reported risky sexual behavior. Participants reported the number of lifetime partners on a 7-point scale (0 ‘0’; 1-2 ‘1’; 3-5 ‘2’; 6-10 ‘3’; 11-15 ‘4’; 16-20 ‘5’; 21-50 ‘6’; 50+ ‘7’). In addition, a risky sexual behavior score was created by multiplying the number of partners in the past 12 months with a sum of two scales: new-partner condom nonuse (0 ‘*no new partner*’; 1 ‘*always use condom*’; 2 ‘*most times use condom*’; 3 ‘*about half time use condom*’; 4 ‘*sometimes nonuse*’; and 5 ‘*never use*’) and regular-partner condom nonuse (1 ‘*always use condom*’; 2 ‘*most times use condom*’; 3 ‘*about half time use condom*’; 4 ‘*sometimes nonuse*’; and 5 ‘*never use*’).

***Any intimate partner violence.*** Self-reports of partner violence were measured with the self-report 47-item General Violence Questionnaire (Holtzworth-Munroe et al., 2000). An indicator was coded 1 if the respondent perpetrated violent acts towards a romantic partner in the past 12 months (i.e., threatened with a knife or gun; pushed, shoved, grabbed, slapped, or threw something; punched, hit, kicked, bit, or slammed against a wall; beat up or choked, strangled, burned, or scalded on purpose; or used a knife or gun).

***Parenting****.* Participants with children completed the Conflict Tactics Scale (Straus, 1979). Coercive parenting ( = 0.79) was constructed by averaging across items capturing five behaviors (threatened; yelled, insulted, or swore at; hit or tried to hit with something; pushed, grabbed, or slapped; and beat up). Two items assessing spanking and threatening to spank were also averaged to create a second scale ( = 0.81). All items were coded as 1 ‘*never*’, 2 ‘*1-3 times*’, 3 ‘*monthly*’, 4 ‘*weekly*’, and 5 ‘*most days*’.

***Criminal offenses.*** Administrative court records were collected using national databases (based on full name, birthdate, and social security number) that included all arrests, adjudications, diversions, and magistrate appearances. We limited offenses to convictions and diversions of violent, substance, and property or public order crime. Severity-weighted indices were created by multiplying frequencies with severity across all lifetime adult convictions (CPPRG, 2010). For violent crimes, severity levels ranged from 1 to 3 (severity 3 included aggravated/armed robbery, murder, rape, kidnapping, sex offenses, and first-degree assault; severity 2 included robbery and first-degree burglary; and severity 1 included DUI and carrying a concealed weapon). Severity levels for substance crimes ranged from 1 to 2 (severity 2 includes manufacturing and possession with intent to sell; severity 1 included possession). Severity levels for property/public order crimes ranged from 1 to 3 (severity 3 included breaking and entering, identity theft, forgery, failure to register as a sex offender, and prostitution; severity 2 included possession of stolen property, vandalism, disorderly conduct, violation of protection order or contempt; severity 1 included loitering, littering, and public consumption). An aggregate index across all violent, substance, property, and public order convictions was also constructed.

**Mechanisms of Action: Elementary and Middle School Skills**

These scores were designed as indices to aggregate the skills intervention youth acquired from 1^st^ through 8^th^ grade in each of these domains (see Godwin & CPPRG, 2020, for further details). Supplementary Table S3 provides a detailed description of each scale included in each composite mechanism of action score.

***Interpersonal skills****.* Interpersonal skills included 15 scores: authority acceptance reported by classroom observers in grade 1 and teachers in grade 3 (reverse coded), parent- and teacher-reported positive child behavior changes in grades 1 and 3, interviewer-rated positive peer interactions in grade 1, peer social preference and peer-nominated prosocial behavior in grade 1, parent report of oppositional and aggressive behavior (reverse coded) in grade 3, indicator for home and community problems (reverse coded) and deviant peer problems (reverse coded) in grades 4 and 5, self-reported index offenses in grade 7 (reverse coded), parent-reported hyperactivity in grade 7 (reverse coded), and deviant activity of best friend in grade 8 (reversed coded).

***Intrapersonal skills****.* Intrapersonal skills included seven child-reported scores: competent social problem solving in grades 1 and 3, emotional recognition in grade 1, emotion coping skills in grade 1, endorsement of aggressive retaliation in grade 1 (reverse coded), hostile attributions in grade 3 (reverse coded), and indicator for social cognition and competence problems in grades 4 and 5 (reverse coded).

***Academic skills****.* Academic skills included four scores: language arts grades, Spache diagnostic reading subscale, minutes of special education services (reverse coded) in first grade, and an indicator for special education diagnosis in third grade (reverse coded).

**Results**

**Total Effects of Intervention**

As seen in Supplementary Table S2, assignment to intervention was significantly associated with few adult outcomes when total effects are examined. Unexpectedly, the odds of being employed full time were 32% lower for intervention participants relative to control participants (*OR*=0.68). While the odds of *never* being convicted of a substance-related crime by age 31 were 34% lower for intervention participants relative to control participants, the expected, severity-weighted substance-related conviction index among those convicted of at least one crime was significantly lower for intervention participants relative to control participants. That is, the expected index was 24% lower for intervention participants convicted of at least one substance-related crime (IRR=0.76) relative to control participants. While the intervention-control difference in the probability of *never* being convicted of a violent crime by age 31 was not significant, the expected, severity-weighted violent conviction index was 24% lower for intervention participants convicted of at least one violent crime (IRR=0.76) relative to control participants. Finally, as mentioned in the main manuscript, when examining mechanisms of action, it appears that Fast Track conveyed numerous beneficial intervention effects on adult outcomes via its improvement of interpersonal and intrapersonal in childhood and adolescence. Further detail concerning these beneficial effects can be found in the Results section of the main manuscript.

**Supplementary Table S1.** Intervention Components (see CPPRG, 2020, for further details.)

| **Elementary School (grades 1-5)** | |
| --- | --- |
| PATHS Curriculum | Teachers taught a grade-level version of the PATHS® (Promoting Alternative Thinking Strategies) Curriculum (Greenberg et al., 2011), which is a social-emotional learning intervention targeting prosocial skills, self-control, emotional awareness and understanding, and social problem solving to increase social and emotional competence. |
| Friendship Groups | Friendship groups targeted children’s prosocial skills, emotional expression and emotional understanding, accurate awareness of others’ intentions, anger control, emotional regulation, and social problem-solving skills. Coaching methods focused on direct instruction and modeling to build skill concepts, behavioral rehearsal, and performance feedback. |
| Peer Pairing | Peer pairing involved a supervised half-hour play session with classroom peers each week that focused on practicing skills introduced in friendship group sessions. |
| Parent Groups | Parent groups aimed to improve positive parent–child interactions, reduce harsh and punitive discipline, and increase consistent limit setting. In addition, these groups also sought to promote the development of positive family-school relationships, enhance parent self-control, and foster parents’ abilities to support child engagement and adjustment at school. |
| Parent-Child Sharing | Parent-child sharing sessions had two primary goals: foster positive parent-child relationships through the promotion of positive, cooperative parent-child interactions; and provide parents with an opportunity to practice the skills introduced in parent groups with staff guidance. |
| Home Visiting | Home visits provided intervention staff an opportunity to meet other family members and better understand the family situation, review the parenting skills within the family context, strengthen parent-school engagement, and address any family issues by referring to other community supports. |
| Academic Tutoring | Using the Wallach reading tutoring program (Wallach & Wallach, 1976), this structured one-on-one tutoring program was designed to serve low-readiness children emphasizing a phonics-based, mastery-oriented approach to the development of initial reading skills. |
| Mentoring | Children were provided with a same-sex, same-race community volunteer mentor to foster the development of personal goals and aspirations for the future and strengthen social support in the child’s community. |
| **Middle and High School (grades 6-10)** | |
| Parent Groups and Youth Forums | These group sessions included discussions regarding school transition pressures, relationships, substance use, and vocational interests. |
| Middle-School Transition Program | During the transition into middle school, students were offered individual or small group sessions to help youth and their parents adjust to the new demands and opportunities of the middle-school context. These sessions included orientation of the middle school, and visits from school counselors, experienced parents, and older peers to provide information about the middle-school experience. |
| Academic Achievement and Orientation | This included tutoring in individual or small group sessions to assist with homework and enhance study skills, behavioral contracting for school performance to increase specific behaviors, assistance in the school setting to promote youths’ attention skills, and consultation with teachers to foster tutoring effectiveness or behavioral management. |
| Peer Relations | This included individual or small group meetings to discuss problems with peers and identify action plans, behavioral contracting for social-behavioral growth to increase specific behaviors, mentoring to provide recreational opportunities and positive models for identity development, and supporting and facilitating youth participation in school or community groups to support positive peer affiliations and engagement. |
| Parenting and Adult Involvement | This included problem-solving meetings with parents focusing on monitoring, limit setting, and communication skills; family problem-solving meetings involving efforts to promote parent-youth relationships and communication; short-term crisis intervention to help parents utilize effective problem-solving skills under stressful circumstances; and referral to community service agencies for parents who experience more chronic crises and require general support. |
| Identity Development | This included individual or small group meetings with youth to provide support for the youths’ development of personal future goals and the acquisition of skills needed to pursue their goals. |

**Supplementary Table S2.** Missing Data Analyses

|  | **Sample with Age 31 Data** | |  | **Sample Missing Data at Age 31** | | **Test Statistic** | **df** | ***p*-value** |
| --- | --- | --- | --- | --- | --- | --- | --- | --- |
|  | ***N*** | ***M (SD)* Proportion** |  | ***N*** | ***M (SD)* Proportion** |  |  |  |
| Intervention* | 657 | 0.50 |  | 234 | 0.49 | 0.19 | 1 | 0.66 |
| Site* |  |  |  |  |  | 15.17 | 3 | 0.00 |
| Durham | 657 | 0.26 |  | 234 | 0.19 |  |  |  |
| Nashville | 657 | 0.23 |  | 234 | 0.33 |  |  |  |
| Seattle | 657 | 0.23 |  | 234 | 0.27 |  |  |  |
| Rural Pennsylvania | 657 | 0.27 |  | 234 | 0.20 |  |  |  |
| Cohort* |  |  |  |  |  | 2.28 | 2 | 0.32 |
| cohort 1 | 657 | 0.36 |  | 234 | 0.32 |  |  |  |
| cohort 2 | 657 | 0.36 |  | 234 | 0.35 |  |  |  |
| cohort 3 | 657 | 0.28 |  | 234 | 0.33 |  |  |  |
| Male* | 657 | 0.66 |  | 234 | 0.78 | 10.58 | 1 | 0.00 |
| Black* | 657 | 0.52 |  | 234 | 0.48 | 1.04 | 1 | 0.31 |
| Initial Screen Score | 640 | -0.01 (0.98) |  | 229 | 0.04 (1.05) | -0.67 | 867 | 0.51 |
| Parental Depression | 656 | 0.02 (1.01) |  | 234 | -0.04 (0.99) | 0.79 | 888 | 0.43 |
| Child Hostile Attribution | 657 | 0.01 (1.00) |  | 233 | -0.04 (1.01) | 0.75 | 888 | 0.46 |
| Child Aggressive Behavior | 656 | 0.00 (1.00) |  | 232 | 0.00 (1.01) | -0.05 | 886 | 0.96 |
| Child Emotional Appropriateness Score | 657 | -0.03 (1.06) |  | 234 | 0.01 (0.82) | -1.91 | 525 | 0.06 |
| Parental Family Satisfaction | 653 | 0.02 (1.01) |  | 231 | -0.04 (0.97) | 0.79 | 882 | 0.43 |
| Parental Friendship Satisfaction | 655 | 0.03 (1.01) |  | 232 | -0.07 (0.97) | 1.29 | 885 | 0.20 |
| Parent-Report of Physical Punishment | 655 | -0.01 (1.02) |  | 234 | 0.02 (0.94) | -0.28 | 887 | 0.78 |
| Parent-Report of Kindergarten Stress | 651 | 0.01 (1.04) |  | 233 | -0.03 (0.89) | 0.55 | 470 | 0.58 |
| Parent-Report of Verbal Punishment | 655 | 0.01 (1.02) |  | 234 | -0.04 (0.94) | 0.65 | 887 | 0.52 |
| Socioeconomic Status | 654 | 0.01 (1.00) |  | 234 | -0.02 (1.00) | 0.41 | 886 | 0.68 |
| Child Oppositional Aggressive Score | 657 | 0.02 (1.01) |  | 231 | -0.05 (0.96) | 0.82 | 886 | 0.41 |
| Parental Harsh Discipline, Warmth, & Appropriate Discipline Score | 653 | 0.00 (1.02) |  | 232 | 0.00 (0.95) | 0.00 | 883 | 1.00 |
| Child Social Competence - Parent Report | 656 | -0.02 (1.03) |  | 234 | 0.05 (0.92) | -0.9 | 453 | 0.37 |
| Child Woodcock-Johnson Word Identification Score | 656 | 0.02 (1.01) |  | 233 | -0.05 (0.96) | 0.98 | 887 | 0.33 |
| Child Emotional Recognition | 647 | 0.02 (1.00) |  | 230 | -0.06 (0.99) | 1.06 | 875 | 0.29 |
| Parental Warmth | 655 | 0.01 (1.01) |  | 233 | -0.02 (0.99) | 0.36 | 886 | 0.72 |
| Child Competent Social Problem Solving Score | 655 | -0.01 (1.00) |  | 232 | 0.02 (1.01) | -0.43 | 885 | 0.67 |
| Parental Neighborhood Satisfaction Score | 656 | -0.02 (1.02) |  | 234 | 0.07 (0.94) | -1.18 | 888 | 0.24 |
| Child Wechsler Intelligence Scale Average Vocab & Block Design | 655 | 0.00 (1.01) |  | 234 | 0.00 (0.98) | 0.00 | 887 | 1.00 |

*Note.* Statistical differences between values for those with and without age 31 data were assessed using *t*-tests with pooled variances with the exceptions noted. *Chi square tests were used to assess statistical differences for categorical variables. **Rejected null of equal variances between those missing and not missing age 31 data, therefore Satterthwaite *t*-tests were used to assess statistical differences.

**Supplementary Table S3.** Components of Mechanisms of Actions

| **Interpersonal Skills** |  |
| --- | --- |
| Gr 1 - Observer Authority Acceptance Scale - reversed | Using the TOCA-R (Werthamer-Larsson et al., 1991) classroom observers reported the frequency with which the child exhibited the following behaviors during four separate 30-minute observation sessions: Taking property, yelling at others, fights, stubbornness, lying, breaking rules, teasing others, breaking things, harming others, and disobedience. Frequency was rated on a 6-point scale from 1 (almost never) to 6 (7+ times). The score was constructed by averaging the behavior frequencies over all observation sessions. |
| Gr 1 - Time in Positive Peer Interaction | Percentage of time children were engaged in positive peer interactions based on in-person classroom observations collected using MOOSES (Tapp et al., 1995). Each child was observed four times for 30 minutes (CPPRG, 1999). |
| Gr 1 - Peer Social Preference | The Sociometric interview was administered to all students in each child's classroom (Coie et al., 1982). All students were asked to nominate peers whom they "most liked" and "least liked." The scale captures the differences between the child's average rating as "most liked" and "least liked." |
| Gr 1 - Peer-Nominated Prosocial | The Sociometric interview was administered to all students in each child's classroom (Coie et al., 1982). All students assessed peers they preferred to engage with based on items such as cooperating, helping others, and sharing. The scale captures the rating for the child among his/her peers. |
| Gr 1 - Parent Rating of Positive Child Behavior Change | Using parent reports from the Post-Intervention Ratings of Child and Parent Change measure (CCPRG, 1999), this scale is an average across six items capturing the parent's perceptions of change in the child's ability to get along with other children, get along with adults, follow rules, follow instructions, calm down, and not fight with other children over the school year. |
| Gr 1 - Teacher Rating of Positive Child Behavior Change | Using teacher reports from the Teacher Post Ratings measure (CPPRG, 1999), this scale is an average across eight items capturing change in prosocial behavior over the school year including a child's ability to calm down, label emotions, show empathy, handle disagreements, join in play, cooperate, play fair, and demonstrate self-esteem. |
| Gr 3 - Parent Rating of Positive Child Behavior Change | Using parent reports from the Post-Intervention Ratings of Child and Parent Change (CCPRG, 1999), this scale is an average across six items capturing the parent's perceptions of change in the child's ability to get along with other children, get along with adults, follow rules, follow instructions, calm down, and not fight with other children over the school year. |
| Gr 3 - Teacher Rating of Positive Child Behavior Change | Using teacher reports from the Teacher Post Ratings measure (CPPRG, 1999), this scale is an average across eight items capturing change in prosocial behavior over the school year including a child's ability to calm down, label emotions, show empathy, handle disagreements, join in play, cooperate, play fair, and demonstrate self-esteem. |
| Gr 3 - Parent-reported Oppositional and Aggressive behavior - reversed | Using parent reports from the Parent Daily Report measure (Chamberlain & Reid, 1987), this scale is an average across items capturing frequency the child exhibited the following behaviors: fighting with siblings, aggressiveness, hitting, teasing, whining, complaining, yelling, noncompliance, defiance, talking back, and pouting. |
| Gr 3 - Teacher Authority Acceptance - reversed | This scale is an average across teacher reports of the frequency the child exhibited the following behaviors from the teacher-reported TOCA-R (Werthamer-Larsson et al., 1991): taking property, yelling at others, fights, stubbornness, lying, breaking rules, teasing others, breaking things, harming others, and disobedience. |
| Gr 4/5 - Indicator for Home and Community Problems - reversed | The home and community problems domain in grades 4 and 5 included parent reports of 15 oppositional-aggressive behavior items from the Parent Daily Report (Chamberlain & Reid, 1987); parent reports of the child's behavior change over the past year based on Parent Ratings of Child Behavior Change instrument (CPPRG, 1999a); child reports of 24 problem behaviors reported on the Things That You Have Done scale (adapted from Elliott et al., 1985); and child reports of any substance use. For each grade the mean across standardized domain scores was created. The average of those two scores was taken and caseness was coded 1 if the child's score was greater than 1 standard deviation above the mean from the normative FT sample (CPPRG, 2000) and coded 0 if it was not greater than 1 standard deviation above the mean. |
| Gr 4/5 - Indicator for Peer Deviance - reversed | The peer deviance domain in grades 4 and 5 included child reports of whether none, some, or most of their friends used substances (five items) and participated in 11 delinquent behaviors based on reports on Things That Your Friends Have Done measure (CPPRG, 2000). The average of the standardized scores from grades 4 and 5 was taken and caseness was coded 1 if the child's score was greater than 1 standard deviation above the mean from the normative FT sample (CPPRG, 2000) and coded 0 if it was not greater than 1 standard deviation above the mean. |
| Gr 7 - SRD Index Offenses - reversed | Youth completed the Self-Report of Delinquency measure (Huizinga & Elliot, 1987) describing the number of times he/she completed various acts of delinquency. This scale is the mean across 13 items describing whether the following kinds of delinquency were ever committed in the past year: setting fires, stealing, using weapons to get money, attacking people with the intent to hurt them, being involved in gang fight, rape, and selling drugs. |
| Gr 7 - Hyperactivity - reversed | Using parent reports from the Parent Daily Report measure (Chamberlain & Reid, 1987), this scale is an average across items capturing frequency the child exhibited the following behaviors: being noisy, running around, and being hyperactive. |
| Gr 8 - Best Friend deviant activity - reversed | Using items from the Self-Report of Close Friends (O’Donnell et al., 1995), this scale is an average across standardized items capturing frequency that the child's best friend engages in five behaviors (gets into trouble with teachers, drinks alcohol, smokes cigarettes, uses marijuana/drugs, and gets into trouble with the police) and dichotomous indicators for whether the child and their best friend have done seven behaviors together in the past year (got in trouble with the police, stolen things, damaged things, been around alcohol/drugs, drank alcohol, used drugs, and sold drugs). |
| **Intrapersonal** |  |
| Gr 1 - Aggressive Retaliation - reversed | As part of the Home Interview with Child measure (Dodge et al., 1990), children were presented four drawings and vignettes depicting ambiguous minor harm situations and four others depicting unsuccessful peer entry situations. The child reported how they would respond and interviewers coded those responses as child doesn't know, do nothing, ask why/ask again, make a command, threaten adult punishment or make a threat, or aggressive retaliation. These scores are the percentage of the responses that were aggressive retaliation. |
| Gr 1 - Emotion Coping | Children completed the Interview on Emotional Experiences (Greenberg & Kusche, 1990) in which children described the kinds of things that made them feel a certain way (happy, sad, angry, and worried) and what they do when they feel that way. Interviewers coded the child's responses as prosocial/competent or aggressive/inept. This scale captures the percentage of responses that were prosocial/competent (CPPRG, 1999). |
| Gr 1 - Emotion Recognition | As part of the Emotion Recognition Questionnaire (Ribordy et al., 1988), children were given 16 scenarios and asked to identify the emotions of the characters in the scenario (happy, sad, mad, or scared). This scale captures the number of emotions correctly identified. |
| Gr 1 - Competent Social Problem Solving | Children completed the Social Problem Solving measure (Dodge et al., 1990) in which eight drawings with corresponding vignettes were presented that depicted social conflict problems and children were asked how to solve the problem. Responses were coded as prosocial/competent or aggressive/inept. This scale captured the percent of prosocial/competent responses. |
| Gr 3 - Competent Social Problem Solving | Children completed the Social Problem Solving measure (Dodge et al., 1990) in which eight drawings with corresponding vignettes were presented that depicted social conflict problems and children were asked how to solve the problem. Responses were coded as prosocial/competent or aggressive/inept. This scale captured the percent of prosocial/competent responses. |
| Gr 3 - Hostile Attributions - reversed | As part of the Home Interview with Child measure (Dodge et al., 1990) children were presented four drawings and vignettes depicting ambiguous minor harm situations and four others depicting unsuccessful peer entry situations. The child reported whether the offender’s intentions were non-hostile or hostile. This score is the percentage of the responses that were deemed hostile. |
| Gr 4/5 - Indicator for Social Cognition and Social Competence Problems - reversed | The social cognition and social competence problems domain in grades 4 and 5 included two scales in each year. Children completed the What Do You Think measure (CPPRG, 2000) in which children were presented with three stories involving a problematic peer situation and three stories involving a problematic interaction with an adult. For each story, children were asked a series of questions designed to measure the child's tendencies toward hostile attributions, aggressive-punitive responses, endorsement of retribution goals, selection of aggressive responses, and anticipated effectiveness of aggressive responses. An average across the five standardized scores created a total social-cognitive problems score. In addition, teachers completed the Social Competence-Teacher measure (CPPRG, 1999) in which teachers rated children's current levels and improvements over the past year in academic competence and prosocial behavior/emotion regulation skills. An average across the four standardized scores created a total social competence problems score capturing current problems in academic competencies, current problems in social competencies, declines in academic competencies in past year, and declines in social competencies in the past year. The average of those four scores was taken and caseness was coded 1 if the child's score was greater than one standard deviation above the mean from the normative FT sample (CCPRG, 2000) and coded 0 if the score was not greater than 1 standard deviation above the mean. |
| **Academic** |  |
| Gr 1 - Language Arts Grades | Grading systems varied across schools with some using standard A-F scales and others using fewer categories (such as satisfactory vs unsatisfactory). Precise grades were collected from administrative school records. A universal scoring protocol using information from each school's grading system was created (CPPRG, 1999). Scoring delineated low, average, and high grades. Low scores included 1 (corresponding to an F) and 4 (corresponding to a D). Average scores included 5, 7, and 9 capturing Cs and satisfactory process). High scores included 10 (corresponding to a B) and 13 (corresponding to an A). |
| Gr 1 - Spache Word Attack | Children completed the Spache Diagnostic Reading Scale which included five individual assessments (Spache, 1981). The word attack score summed scores on three tests capturing Recognition of Initial Consonants, Recognition of Final Consonants, and Auditory Recognition of Initial Consonant. |
| Gr 1 - Special Education Services (min/wk) - reversed | Number of minutes per week spent in special education based on information collected from administrative school records (CPPRG, 1999). |
| Gr 3 - Special Education Diagnosis - reversed | Indicator for receiving any special education services including a diagnosis, spending time in special education, or receiving an Individualized Education Plan based on information collected from administrative school records (CPPRG, 2002). |

**Supplementary Table S4.** Total Intervention Effects

|  | **Total Intervention Effect (95% CI)** | | |
| --- | --- | --- | --- |
|  | **Est** | ***OR*** | ***IRR*** |
| **Any Externalizing, Internalizing, or Substance Use Problem ^a^** |  | 1.05 (0.78, 1.41) |  |
|  |  |  |  |
| **Externalizing Problems *T*-score** | 0.27 (-1.34, 1.89) |  |  |
| Antisocial Personality DSM Clinical Range^a^ |  | 1.34 (0.79, 2.30) |  |
| ADHD Problems DSM Clinical Range^a^ |  | 1.18 (0.58, 2.38) |  |
|  |  |  |  |
| **Internalizing Problems *T*-score** | -0.81 (-2.71, 1.09) |  |  |
| Avoidant Personality DSM Clinical Range^a^ |  | 0.69 (0.39, 1.21) |  |
| Somatic DSM Clinical Range^a^ |  | 1.07 (0.66, 1.73) |  |
| Anxiety DSM Clinical Range^a^ |  | 0.82 (0.43, 1.57) |  |
| Depression DSM Clinical Range^a^ |  | 1.08 (0.59, 1.95) |  |
|  |  |  |  |
| **Any Problematic Substance Use^a^** |  | 1.05 (0.75, 1.46) |  |
| Binge Drinking Problem^a^ |  | 0.83 (0.45, 1.55) |  |
| Heavy Cannabis Use^a^ |  | 1.13 (0.74, 1.74) |  |
| Serious Substance Use^a^ |  | 0.74 (0.44, 1.22) |  |
|  |  |  |  |
| **Overall Wellbeing** | -0.01 (-0.11, 0.09) |  |  |
| General Health Index | -0.01 (-0.04, 0.02) |  |  |
| Happiness Score | 0.15 (-0.64, 0.94) |  |  |
| Strength Score | -0.09 (-0.68, 0.50) |  |  |
|  |  |  |  |
| **Education and Employment** |  |  |  |
| High School Diploma/GED^a^ |  | 1.18 (0.78, 1.80) |  |
| Currently Full-time Employed^a^ |  | 0.68 (0.49, 0.94)* |  |
|  |  |  |  |
| **Romantic Relationships** |  |  |  |
| Number of Sexual Partners over Lifetime | -0.08 (-0.40, 0.23) |  |  |
| Risky Sexual Behavior in Past 12 Months | 0.89 (-4.06, 5.83) |  |  |
| Any Intimate Partner Violence^a^ |  | 1.13 (0.71, 1.82) |  |
|  |  |  |  |
| **Parenting** |  |  |  |
| Coercive Parenting | 0.03 (-0.04, 0.10) |  |  |
| Spanking Scale | -0.02 (-0.20, 0.16) |  |  |
|  |  |  |  |
| **Severity-Weighted Conviction Indices** |  |  |  |
| All Crime^b^ |  | 0.94 (0.67, 1.31) | 0.89 (0.76, 1.05) |
| Substance Crime^b^ |  | 0.66 (0.44, 0.98)* | 0.76 (0.59, 0.96)* |
| Violent Crime^b^ |  | 0.96 (0.68, 1.34) | 0.76 (0.58, 0.99)* |
| Property & Public Order Crime^b^ |  | 0.97 (0.73, 1.29) | 0.96 (0.76, 1.20) |

^a^ Outcome is dichotomous and the intervention effect was modeled using a logistic regression and presented as an *OR*. ^b^ Outcome is a count variable and the intervention effect was modeled using a zero-inflated Poisson regression with the impact of intervention on the probability of a structural zero presented as an *OR* and the intervention effect on the expected count among those with a non-zero value presented as an IRR. Complete set of results are available upon request. **p*<.05

**Supplementary Figure S1:** Pathway of Fast Track Mechanisms of Action


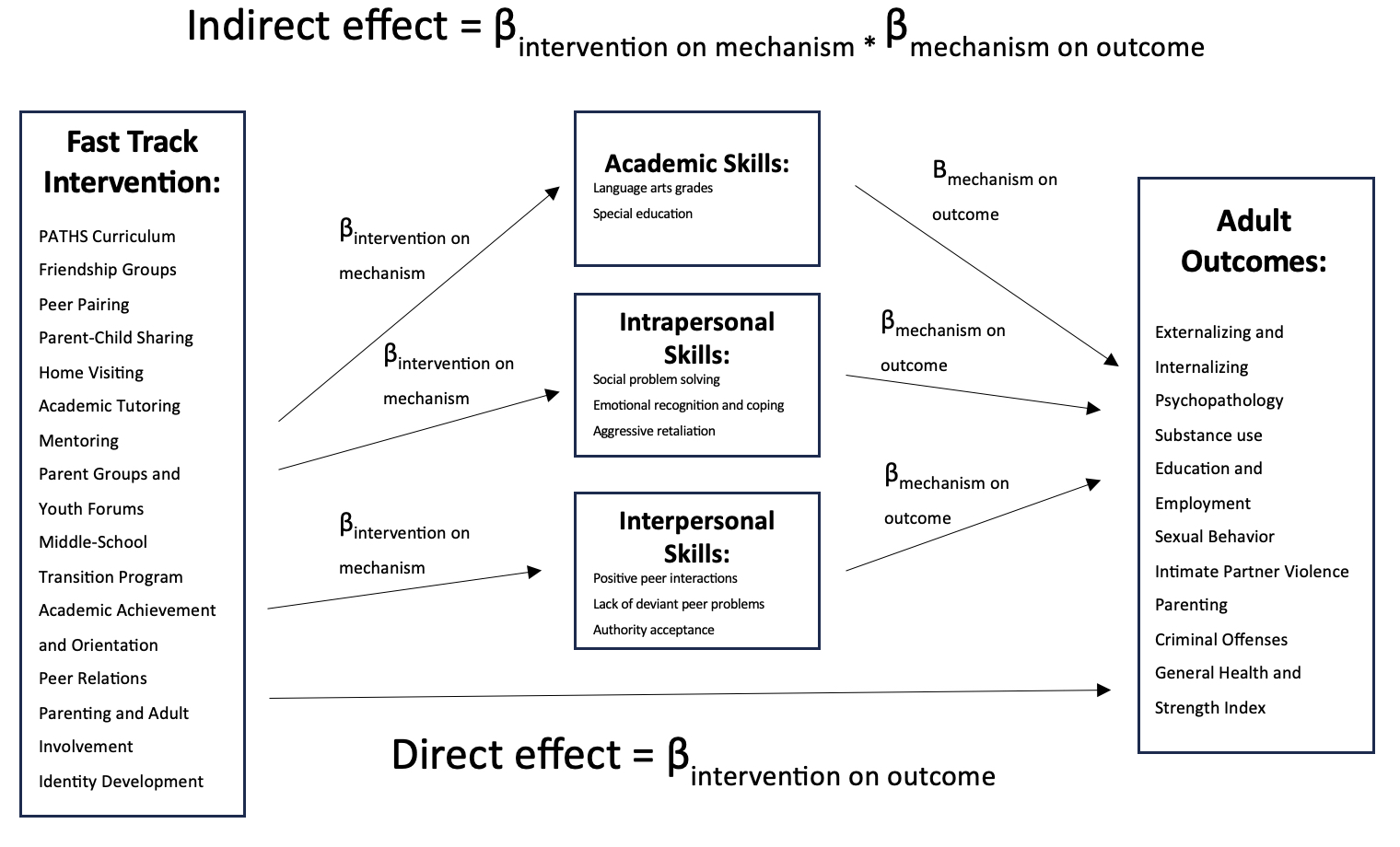


**References**

Achenbach, T. M. (1997). *Manual for the Young Adult Self-Report and Young Adult Behavior Checklist*. University of Vermont, Department of Psychiatry.

Bureau of Labor Statistics, US Department of Labor. (2002). *National Longitudinal Survey of Youth 1997 Cohort, 1997–2001*. US Department of Labor, Bureau of Labor Statistics.

Capaldi, D. M., Stoolmiller, M., Clark, S., & Owen, L. D. (2002). Heterosexual risk behaviors in at-risk young men from early adolescence to young adulthood: Prevalence, prediction, and association with STD contraction. *Developmental Psychology*, *38*(3), 394-406. <https://doi.org/10.1037/0012-1649.38.3.394>

Chamberlain, P. & Reid, J. B. (1987). Parent observation and report of child symptoms. *Behavioral Assessment*, *9*, 97-109.

Coie, J. D., Dodge, K. A., & Coppotelli, H. A. (1982). Dimensions and types of social status: A cross-age perspective. *Developmental Psychology, 18*(4), 557-570. <https://doi.org/10.1037/0012-1649.18.4.557>

Conduct Problems Prevention Research Group. (1999). Initial impact of the Fast Track prevention trial for conduct problems: I. The high-risk sample. *Journal of Consulting and Clinical Psychology 67*(5), 631-641*.* <https://doi.org/10.1037/0022-006X.67.5.631>

Conduct Problems Prevention Research Group. (2010). Fast Track intervention effects on youth arrests and delinquency. *Journal of Experimental Criminology*, *6*(2), 131-157. <https://doi.org/10.1007/s11292-010-9091-7>

Conduct Problems Prevention Research Group. (2020). *The Fast Track program for children at risk: Preventing antisocial behavior*. Guilford Press.

Dodge, K. A., Bates, J. A., & Pettit, G. S. (1990). Mechanisms in the cycle of violence. *Science, 250*(4988)*,* 1678-1683. <https://doi.org/10.1126/science.2270481>

Elliott, D. S., Huizinga, D., & Ageton, S. S. (1985). *Explaining delinquency and drug use*. Sage.

Greenberg, M. T., & Kusche, C. A. (1990) *Inventory of Emotional Experience: Technical report.* Seattle: University of Washington.

Greenberg, M. T., Kusche, C. A., & CPPRG. (2011). *Grade level PATHS (Grades 3-4).* Channing-Bete.

Holtzworth-Munroe, A., Rehman, U., & Herron, K. (2000). General and spouse-specific anger and hostility in subtypes of maritally violent men and nonviolent men. *Behavior Therapy*, *31*(4), 603-630. <https://doi.org/10.1016/S0005-7894(00)80034-9>

Howe, D., & Frazis, D. (1992). *What researchers have learned from the National Longitudinal Surveys about Youth Unemployment (no. 828)*. US Department of Labor, Bureau of Labor Statistics.

Huizinga, D., & Elliott, D. S. (1987). Juvenile offenders: Prevalence, offender incidence, and arrest rates by race. *Crime & Delinquency, 33*(2), 206-223. <https://doi.org/10.1177/001112878703300202>

O’Donnell, J., Hawkins, J. D., & Abbott, R. D. (1995). Predicting serious delinquency and substance use among aggressive boys. *Journal of Consulting and Clinical Psychology*, 63, 529-537.

Ribordy, S. C., Camras, L. A., Stefani., & Spaccarelli, S. (1988). Vignettes for emotion recognition research and affective therapy with children. *Journal of Clinical Child Psychology, 17*(4)*,* 322-325. <https://doi.org/10.1207/s15374424jccp1704_4>

Seligman, M. E. P. (2011). *Flourish: A visionary new understanding of happiness and well-being*. Free Press.

Spache, G. D. (1981). *Diagnostic Reading Scales Examiner's Manual*. CTB/McGraw-Hill.

Tapp, J. T., Wehby, J. H., & Ellis, D. N. (1995). A multiple option observation system for experimental studies: MOOSES. *Behavior Research Methods, Instruments and Computers, 27*(1), 25-31. <https://doi.org/10.3758/BF03203616>

Wallach, M. A., & Wallach, L. (1976). *Teaching all children to read*. University of Chicago Press.

Ware, J. E., & Sherbourne, C. D. (1992). The MOS 36-item Short-Form Health Survey (SF-36): I. Conceptual framework and item selection. *Medical Care*, *30*(6)*,* 473-483.

Werthamer-Larsson, L., Kellam, S. G., & Wheeler, L. (1991). Effect of first-grade classroom environment on shy behavior, aggressive behavior, and concentration problems. *American Journal of Community Psychology, 19*(4), 585-602. <https://doi.org/10.1007/BF00937993>
